# Pulmonary Hypertension in Down Syndrome Versus Non-syndromic Pediatric Populations With Congenital Heart Disease: A Comparative Study

**DOI:** 10.7759/cureus.104634

**Published:** 2026-03-03

**Authors:** Md Tariqul Islam, Tahmina Karim, Sadia Afrin Mony, Md Mostafizur Rahman Bhuyan, Faizah Islam

**Affiliations:** 1 Pediatric Cardiology, Bangladesh Medical University, Dhaka, BGD; 2 Cardiology, Bangladesh Medical University, Dhaka, BGD

**Keywords:** atrioventricular septal defect (avsd), congenital heart disease (chd), down syndrome, echocardiography, non-syndromic chd, pediatric cardiology, pulmonary hypertension (ph), systolic pulmonary artery pressure (spap), tricuspid annular plane systolic excursion (tapse)

## Abstract

Background: Down syndrome (DS), or trisomy 21, is the most common chromosomal disorder associated with congenital heart disease (CHD), profoundly affecting disease progression and management. While 4-10% of all CHD cases occur in DS, 40-60% of individuals with DS have CHD. CHD remains the leading cause of mortality in DS within the first two years of life, with atrioventricular septal defect (AVSD), ventricular septal defect (VSD), atrial septal defect (ASD), patent ductus arteriosus (PDA), and tetralogy of Fallot (TOF) being most prevalent. Pulmonary hypertension (PH) frequently complicates these lesions, influencing prognosis and therapeutic decisions. Early detection via echocardiography is crucial for optimising outcomes. This study compares the prevalence and phenotypic patterns of pulmonary hypertension in paediatric patients with syndromic versus non-syndromic congenital heart disease.

Materials and methods: A cross-sectional study was conducted over 24 months in the Paediatric Cardiology Department of Bangladesh Medical University (BMU), enrolling 208 children with CHD, including 104 with DS and 104 non-syndromic controls between 2022 and 2024. Karyotyping confirmed DS, while 2D, M-mode, and colour Doppler echocardiography diagnosed CHD and assessed pulmonary artery pressure (PAP). Statistical analysis was performed using SPSS version 26 (IBM Corp., Armonk, New York, USA); categorical variables were compared with χ² tests and continuous variables with unpaired t-tests, with p < 0.05 considered significant.

Results: Children with DS presented earlier (2.02 ± 3.77 vs. 3.78 ± 3.8 years; p < 0.001) and had a higher prevalence of PH (97 (88.5%) vs. 55 (52.9%)). Severe PH (>70 mmHg) was more common in DS (18 (17.3%) vs. 7 (6.7%)), while normal PAP was rare (9 (8.7%) vs. 40 (38.5%); p < 0.001). Echocardiography revealed lower tricuspid annular plane systolic excursion (TAPSE) (15.7 ± 3.71 mm vs. 18.3 ± 3.77 mm; p < 0.001) and higher pulmonary artery systolic pressure (PASP) (48.9 ± 16.9 mmHg vs. 35.2 ± 14.4 mmHg; p < 0.001). AVSD predominated in DS (36 (34.6%)), associated with moderate-severe PH, whereas VSD and ASD were common in non-syndromic children, typically with mild or no PH. Combined lesions correlated with higher PH severity in both groups.

Conclusion: Children with DS and CHD exhibit earlier onset and greater severity of PH than non-syndromic peers. Prompt diagnosis and tailored management are critical to prevent progression to advanced pulmonary vascular disease in this high-risk population.

## Introduction

Down syndrome (DS), or trisomy 21, is the most common chromosomal abnormality, affecting approximately one in 700 live births [1]. Life expectancy in individuals with DS is markedly reduced, largely due to cardiopulmonary comorbidities [2,3]. Pulmonary hypertension (PH) is a serious complication, particularly in children with congenital heart disease (CHD), and substantially increases morbidity and mortality in this population [4-7]. CHD occurs in 38-58% of children with DS, most frequently atrioventricular septal defect (AVSD), ventricular septal defect (VSD), atrial septal defect (ASD), patent ductus arteriosus (PDA), and tetralogy of Fallot (TOF) [5]. Many of these lesions produce left-to-right shunts, resulting in pulmonary overcirculation, vascular remodelling, raised pulmonary vascular resistance, and subsequent PH [1,8]. PH also arises in DS patients without CHD, suggesting additional respiratory and cardiovascular mechanisms [9], with prevalence estimates ranging from 6% at one year to 15% by ten years of age.

PH often progresses silently, particularly in children with limited activity or those unable to perform formal functional assessments. Hemodynamically, it is defined as a mean pulmonary arterial pressure (mPAP) >25 mmHg at rest according to the 2015 European Society of Cardiology (ESC) guidelines, with echocardiography commonly used to assess severity [10-12]. The sixth World Symposium on Pulmonary Hypertension (WSPH) categorises PH into five groups, with Group 1 encompassing pulmonary arterial hypertension (PAH) and Group 3 including PH secondary to lung disease or hypoxia, frequently observed in DS [13-17]. Children with DS often experience chronic respiratory disorders, such as obstructive sleep apnoea (OSA), recurrent pneumonia, aspiration, and intermittent hypoxia, all contributing to PH via hypoxic pulmonary vasoconstriction [13,14]. Congenital airway anomalies-including tracheobronchomalacia, subglottic stenosis, tracheal bronchus (3-5%), and laryngeal clefts (1%)-further exacerbate pulmonary vascular stress [15,16]. In DS patients without CHD, PH is predominantly classified as WSPH Group 3, emphasising the role of pulmonary disease. Insofar as existing literature indicates, a matched non-syndromic congenital heart disease cohort is an appropriate comparator, as it controls for cardiac anatomy, haemodynamic burden, and treatment exposure, thereby isolating the independent contribution of Down syndrome-related genetic, developmental, and pulmonary vascular factors to the risk and phenotype of pulmonary hypertension [11-16].

Despite extensive study in developed countries, comparative data on the prevalence, onset, and patterns of pulmonary hypertension in syndromic versus non-syndromic paediatric congenital heart disease remain limited in the Bangladeshi population. This study compared the prevalence and phenotypic patterns of pulmonary hypertension in syndromic versus non-syndromic paediatric populations with congenital heart disease, to inform earlier detection and optimised, individualised management strategies.

## Materials and methods

This cross-sectional comparative study was conducted at the Department of Paediatric Cardiology, Bangladesh Medical University (BMU), previously known as BSMMU, Dhaka, Bangladesh, with data collection spanning from January 2022 to December 2024. The study population comprised children diagnosed with congenital heart disease (CHD), including both syndromic (Down syndrome) and non-syndromic cases, from similar geographic areas to control for environmental and socioeconomic factors. Sample size was determined using Morgan’s table for broad diagnostic categories, yielding 208 participants: 104 children with CHD and Down syndrome, and 104 age- and CHD type-matched children without Down syndrome. Inclusion required confirmed CHD, with Down syndrome verified via karyotyping; critically ill children or those without parental consent were excluded. The primary outcome was pulmonary hypertension, with independent variables including CHD type, age, sociodemographic factors, comorbidities in children with Down syndrome, echocardiographic findings, and CHD anatomy.

Comprehensive sociodemographic data were obtained from the children's legal guardians, ideally their parents, by the principal investigator to ensure uniformity and consistency in data collection. CHD diagnosis and assessment were performed via transthoracic echocardiography (2D, M-mode, pulsed wave, continuous wave, and colour Doppler), confirmed by an expert paediatric cardiologist. Left ventricular ejection fraction (LVEF) was calculated using Simpson's method, with ≥50% considered normal. Diastolic function was assessed via Doppler imaging (E/A ratio, deceleration time, isovolumetric relaxation time) and categorised according to established criteria. Right ventricular function was evaluated using tricuspid annular plane systolic excursion (TAPSE) and tricuspid S wave velocity (tissue Doppler). Systolic pulmonary artery pressure (SPAP) was estimated from tricuspid regurgitant jet velocity using the modified Bernoulli equation; in ventricular septal defect (VSD) or patent ductus arteriosus (PDA), SPAP was adjusted by shunt gradients. Mean pulmonary artery pressure (mPAP) value >25 mmHg is defined as pulmonary hypertension. The severity of pulmonary hypertension was defined according to standard guidelines: mPAP values of 25-49 mmHg were classified as mild, 50-69 mmHg as moderate, and values greater than 70 mmHg as severe PH [10,11]. Pulmonary-to-systemic flow ratio (Qp/Qs) and structural cardiac anomalies (situs, atrioventricular and ventriculoarterial concordance, shunts, valve lesions) were also documented. Down syndrome was confirmed cytogenetically via standard karyotyping procedures. Additionally, the selected cut-off values in this study are based on established echocardiographic thresholds: a TAPSE <16 mm reflects impaired right ventricular systolic function; a pulmonary artery acceleration time (PACT) <90 ms indicates elevated pulmonary vascular resistance and early pulmonary hypertension [10,11]. A PASP of 35 mmHg or greater is widely accepted as indicative of pulmonary hypertension in paediatric echocardiographic practice, thereby ensuring clinical relevance and facilitating comparability with previous studies. Operational definitions included: CHD as a structural heart abnormality present from birth, cyanosis as bluish skin or mucosa with ≥5 g/dL reduced haemoglobin, cardiac murmur as any abnormal heart sound on auscultation, and Down syndrome as trisomy 21 confirmed via karyotyping. This study utilises a matched non-syndromic CHD cohort that provides an appropriate comparator because it controls for cardiac anatomy and disease severity, allowing the independent effect of Down syndrome on outcomes to be isolated while minimising confounding from underlying congenital heart defects.

Ethical approval was obtained from the Bangladesh Medical University Institutional Review Board (BSMMU/2022/10369). Written informed consent was secured from all parents or guardians. Participation was voluntary, confidential, and posed no risk to children; no therapeutic interventions were administered.

Data were processed in IBM SPSS Statistics version 26 (IBM Corp., Armonk, New York, USA). Descriptive statistics summarised demographic and clinical variables. Categorical data were expressed as frequencies and percentages; continuous data as means±SD. Chi-square tests compared categorical variables, and unpaired t-tests compared continuous variables. Univariate analysis explored associations between risk factors and pulmonary hypertension, and multicollinearity among independent variables was assessed using variance inflation factors (VIFs) and collinearity tolerance, with VIF >10 or tolerance <0.1 indicating significant multicollinearity requiring adjustment. Statistical significance was set at p < 0.05.

## Results

Children with congenital heart disease (CHD) and Down syndrome were younger than those without Down syndrome (mean age 2.02±3.77 years vs. 3.78±3.80 years, p < 0.001), and a higher proportion were under one year of age (51 (49%) vs. 35 (33.7%)) (Table 1). No significant differences were identified in the distribution of female sex (61 (58.7%) vs. 57 (54.8%); p=0.904) or parental consanguinity (12 (11.5%) vs. 11 (10.6%); p=0.825).

**Table 1:** Comparison of age, sex, and consanguinity in children with congenital heart disease (CHD) with and without Down syndrome (N=208). Note: Values are shown as N (%) or mean±SD. P-values marked with '*' indicate statistical significance. A p-value obtained by the unpaired t-test, chi-square test, and Fisher's exact test if the sample size is <5 for categorical variables. A p-value of <0.05 was considered significant.

Variables	CHD with Down syndrome (n=104)	CHD without Down syndrome (n=104)	p-value	Test statistics
Age group				
<1 year	51 (49.0%)	35 (33.7%)	<0.001*	χ^2^=21.55
1-5 years	47 (45.2%)	40 (38.5%)		
6-10 years	4 (3.8%)	21 (20.2%)		
11-15 years	0 (0.0%)	8 (7.7%)		
>15 years	2 (1.9%)	0 (0.0%)		
Total	104 (100.0%)	104 (100.0%)		
Mean±SD	2.02±3.77	3.78±3.8	<0.001*	t=3.353
Sex				
Male	43 (41.3%)	47 (45.2%)	0.904	χ^2^=0.313
Female‎	61 (58.7%)	57 (54.8%)		
Consanguinity				
Yes	12 (11.5%)	11 (10.6%)	0.825	χ^2^=0.049
No	92 (88.5%)	93 (89.4%)		

Children with Down syndrome exhibited lower tricuspid annular plane systolic excursion (TAPSE) (15.7±3.71 vs. 18.3±3.77; p < 0.001) and higher pulmonary artery systolic pressure (PASP) (48.9±16.9 vs. 35.2±14.4; p < 0.001), indicating impaired right ventricular function and a higher prevalence of pulmonary hypertension (Table 2). Pulmonary artery capacitance (PACCT) was slightly lower in the Down syndrome group, but this difference was not statistically significant (97.7±17.7 vs. 102.3±20.2; p=0.084).

**Table 2:** Comparison of TAPSE, PACT, and PASP between CHD patients with and without Down syndrome (N=208). TAPSE: tricuspid annular plane systolic excursion; PACT: pulmonary artery acceleration time; PASP: pulmonary artery systolic pressure; CHD: congenital heart disease. Values are presented as N (%) or mean±standard deviation (SD). P-values marked with '*' denote statistical significance. P-values were calculated using the unpaired t-test for continuous variables and the chi-square test or Fisher's exact test for categorical variables with sample sizes less than 5. A p-value less than 0.05 was considered statistically significant.

Variables	CHD with Down syndrome (n=104)	CHD without Down syndrome (n=104)	p-value	Test statistics
TAPSE				
>16 mm	64 (61.5%)	87 (83.7%)	<0.001*	χ^2^=12.784
<16 mm	40 (38.5%)	17 (16.3%)		
Mean±SD	15.7±3.71	18.3±3.77	<0.001*	t=5.013
PACT				
>90 ms	75 (72.1%)	83 (79.8%)	0.10	χ^2^=1.658
<90 ms	29 (27.9%)	21 (20.2%)		
Mean±SD	97.7±17.7	102.3±20.2	0.084	t=1.747
PASP				
>35 mmHg	92 (88.5%)	55 (52.9%)	<0.001*	χ^2^=31.755
<35 mmHg	12 (11.5%)	49 (47.1%)		
Mean±SD	48.9±16.9	35.2±14.4	<0.001*	t=6.293

The distribution of congenital heart disease types differed significantly between groups (Table 3). Atrioventricular septal defect (AVSD) was common in children with Down syndrome (36 (34.6%) vs. 2 (1.9%); p < 0.001), while isolated ventricular septal defect (VSD) (18 (17.3%) vs. 34 (32.7%); p < 0.001), atrial septal defect (ASD) (16 (15.4%) vs. 33 (31.7%); p < 0.001), and patent ductus arteriosus (PDA) (9 (8.7%) vs. 19 (18.3%); p < 0.001) were more prevalent in non-syndromic children.

**Table 3:** Comparison of types of congenital heart disease in children with and without Down syndrome (N=208). AVSD: atrioventricular septal defect; VSD: ventricular septal defect, ASD: atrial septal defect; CHD: congenital heart disease; PDA: patent ductus arteriosus. Values are shown as N (%), and P-values marked with '*' indicate statistical significance. P-value obtained by the chi-square test and Fisher's exact test if the sample size <5. A p-value of <0.05 was considered significant.

Type of congenital heart disease	CHD with Down syndrome (n=104)	CHD without Down syndrome (n=104)	p-value	Test statistics
AVSD	36 (34.6%)	2 (1.9%)	<0.001*	χ^2^=50.68
VSD	18 (17.3%)	34 (32.7%)		
ASD	16 (15.4%)	33 (31.7%)		
PDA	9 (8.7%)	19 (18.3%)		
ASD+VSD	4 (3.8%)	7 (6.7%)		
ASD+PDA	13 (12.5%)	5 (4.8%)		
VSD+PDA	5 (4.8%)	3 (2.9%)		
ASD+VSD+PDA	3 (2.9%)	1 (1.0%)		

The severity of pulmonary hypertension also differed significantly between the study groups (Table 4). Only 9 (8.7%) children with Down syndrome had normal pulmonary pressures, compared to 40 (38.5%) of non-syndromic children. Additionally, severe pulmonary hypertension was more frequent in the Down syndrome group than in those with CHD without Down syndrome (18 (17.3%) vs. 7 (6.7%); p < 0.001), indicating a higher disease burden.

**Table 4:** Comparison of the severity of pulmonary hypertension in children with CHD with and without Down syndrome (N=208). CHD: congenital heart disease. Note: Values are presented as N (%). P-values marked with '*' indicate statistical significance. P-values were obtained using the chi-square test. A p-value less than 0.05 was considered statistically significant.

Severity of pulmonary hypertension	CHD with Down syndrome (n=104)	CHD without Down syndrome (n=104)	p-value	Test statistics
Normal (<25 mmHg)	9 (8.7%)	40 (38.5%)	<0.001*	χ^2^=27.46
Mild (25-49 mmHg)	60 (57.7%)	45 (43.3%)		
Moderate (50-69 mmHg)	17 (16.3%)	12 (11.5%)		
Severe (≥70 mmHg)	18 (17.3%)	7 (6.7%)		

Analysis of pulmonary hypertension (PH) severity by CHD type demonstrated that AVSD in children with Down syndrome was significantly associated with severe PH (12 (33.3%) vs. 9 (25.0%), p=0.002) compared to the moderate PH population (Table 5). Non-syndromic children exhibited a more heterogeneous CHD pattern, with PH predominantly in the normal or mild range. AVSD was rare in non-syndromic children, with only 2 (1.92%) cases, while VSD (34 (32.69%)) and ASD (33 (31.73%)) were comparatively common. Overall, among children with CHD without Down syndrome, severe PH occurred in 7 (6.7%) cases, while moderate and mild PH were observed in 12 (11.5%) and 45 (43.3%) cases, respectively (p < 0.001). 

Assessment of multicollinearity revealed no significant correlations among the independent variables. This was further confirmed using variance inflation factors (VIFs) and collinearity tolerance, with VIF values <10 and tolerance >0.1 indicating the absence of significant multicollinearity requiring corrective measures.

**Table 5:** Association between CHD type and PH severity in children with and without Down syndrome (N=208). CHD: congenital heart disease; PH: pulmonary hypertension; AVSD: atrioventricular septal defect; VSD: ventricular septal defect, ASD: atrial septal defect; PDA: patent ductus arteriosus. Values are reported as N (%). A hyphen symbol (-) in the empty cells indicates that there is nothing to mention. The p-value was calculated using the chi-square test or Fisher’s exact test for categorical variables with sample sizes less than 5. A p-value less than 0.05 (*) was considered statistically significant.

	Severity of PH				p-value	Test statistics
	Normal (<25)	Mild (25-49)	Moderate (50-69)	Severe (>70)		
CHD with Down syndrome (n=104)	(n=9)	(n=60)	(n=17)	(n=18)	-	
AVSD (n=36)	1 (2.8%)	14 (38.9%)	9 (25.0%)	12 (33.3%)	0.002*	χ^2^=44.84
VSD (n=18)	4 (22.2%)	13 (72.2%)	0 (0.0%)	1 (5.6%)		
ASD (n=16)	2 (12.5%)	13 (81.3%)	1 (6.3%)	0 (0.0%)		
PDA (n=9)	1 (11.1%)	5 (55.6%)	2 (22.2%)	1 (11.1%)		
ASD+VSD (n=4)	0 (0.0%)	2 (50.0%)	0 (0.0%)	2 (50.0%)		
ASD+PDA (n=13)	1 (7.7%)	10 (76.9%)	2 (15.4%)	0 (0.0%)		
VSD+PDA (n=5)	0 (0.0%)	2 (40.0%)	3 (60.0%)	0 (0.0%)		
ASD+VSD+PDA (n=3)	0 (0.0%)	1 (33.3%)	0 (0.0%)	2 (66.7%)		
CHD without Down syndrome (n=104)	(n=40)	(n=45)	(n=12)	(n=7)	-	
AVSD (n=2)	0 (0.0%)	1 (50.0%)	0 (0.0%)	1 (50.0%)	<0.001*	χ^2^=52.83
VSD (n=34)	14 (41.2%)	12 (35.3%)	6 (17.6%)	2 (5.9%)		
ASD (n=33)	16 (48.5%)	16 (48.5%)	1 (3.0%)	0 (0.0%)		
PDA (n=19)	9 (47.4%)	9 (47.4%)	1 (5.3%)	0 (0.0%)		
ASD+VSD (n=7)	0 (0.0%)	4 (57.1%)	1 (14.3%)	2 (28.6%)		
ASD+PDA (n=5)	1 (20.0%)	3 (60.0%)	1 (20.0%)	0 (0.0%)		
VSD+PDA (n=3)	0 (0.0%)	0 (0.0%)	2 (66.7%)	1 (33.3%)		
ASD+VSD+PDA (n=1)	0 (0.0%)	0 (0.0%)	0 (0.0%)	1 (100.0%)		

## Discussion

This cross-sectional study compared the prevalence and characteristics of pulmonary hypertension in children with congenital heart disease with and without Down syndrome, demonstrating that those with Down syndrome were diagnosed at a significantly younger age than non-syndromic children, consistent with previous reports indicating earlier detection in this population [18,19]. No significant differences were observed in sex distribution or parental consanguinity between groups (p=0.904 and p=0.825, respectively), supporting prior findings that these factors do not influence CHD prevalence in Down syndrome [20,21].

Right ventricular function was significantly lower, and pulmonary artery pressure was higher, in children with Down syndrome and CHD. TAPSE was reduced (<16 mm) in 38.5% of syndromic children versus 16.3% in non-syndromic children, while 88.5% of the Down syndrome cohort had PASP >35 mmHg compared to 52.9% in non-syndromic children (p < 0.001) [18,20,22]. These findings underline the higher prevalence and severity of PH in Down syndrome, highlighting the utility of TAPSE and PASP as early markers of right heart strain and supporting routine echocardiographic surveillance. Regarding CHD type, atrioventricular septal defect (AVSD) predominated in children with Down syndrome (34.6%), whereas ventricular septal defect (VSD, 32.7%) and atrial septal defect (ASD, 31.7%) were more common in non-syndromic children [5,19,21]. This aligns with the established link between trisomy 21 and endocardial cushion defects, while simpler septal lesions prevail in the general CHD population.

PH severity was markedly greater in Down syndrome, with only 8.7% demonstrating normal pulmonary pressures versus 38.5% in non-syndromic children, and severe PH (≥70 mmHg) observed in 17.3% versus 6.7%, respectively (p < 0.001) [23-27]. AVSD was strongly associated with severe PH, with one-third of cases progressing to severe elevations, whereas simpler defects were generally linked to mild PH or normal pressures [5,19-22]. These findings confirm that children with Down syndrome, particularly those with AVSD, are at heightened risk of early and severe PH, emphasising the importance of timely diagnosis and intervention to prevent irreversible pulmonary vascular disease and improve outcomes.

Overall, this study reinforces the need for early and routine echocardiographic assessment in children with Down syndrome and complex CHDs, particularly AVSD, to facilitate early recognition and management of PH. This study has several notable strengths. First, it employed a structured, comparative design with a clearly defined cohort of 208 children with CHD, equally divided between those with and without Down syndrome, enhancing the reliability of intergroup comparisons. The use of standardised diagnostic criteria, including karyotyping to confirm Down syndrome and comprehensive echocardiography (2D, M-mode, and colour Doppler) to assess CHD and pulmonary hypertension, ensured objective and reproducible measurements. The study also captured data from a single geographic area, reducing potential confounding by regional variations in healthcare access or environmental factors. Additionally, the analysis incorporated well-established echocardiographic markers, such as TAPSE and PASP, strengthening the clinical relevance of the findings and their applicability for early risk stratification. However, several limitations should be acknowledged. Being cross-sectional, the study cannot establish causal relationships or assess the progression of PH over time. The single-centre design may limit generalisability to broader populations with differing genetic, environmental, or healthcare factors. Moreover, reliance on echocardiography without invasive confirmation of pulmonary pressures may introduce measurement variability. Finally, potential confounding factors, such as comorbidities, medical therapy, and socioeconomic influences, were not fully controlled, which could affect the observed associations. Although a multivariable regression model would have enabled estimation of β-coefficients for different congenital heart disease (CHD) subtypes and shunt magnitude, this analysis was not undertaken because the limited number of observations per independent variable would have resulted in inadequate residual degrees of freedom, unstable coefficient estimates, and unreliable statistical inference. Although no significant collinearity was observed, an age-stratified comparison across CHD subtypes could have reduced confounding; however, the small sample sizes within subgroups would have resulted in insufficient statistical power and unstable estimates.

Further evaluation of non-cardiac contributors to pulmonary hypertension in children with Down syndrome may provide additional insight into their impact alongside congenital heart disease. Future studies with larger, adequately powered cohorts are therefore required to permit robust multivariable modelling and to better quantify the independent effects of CHD phenotype and shunt burden on outcomes. A limitation of this study is that pulmonary hypertension was defined using the 2015 ESC criteria (mPAP >25 mmHg). Since the sixth World Symposium on Pulmonary Hypertension (2018) and subsequent updated guidelines have lowered the diagnostic threshold to mPAP >20 mmHg, some patients with mild pulmonary hypertension may not have been captured in our cohort, potentially leading to underestimation of disease prevalence and affecting comparability with more recent studies. Additionally, PASP and mPAP are not directly interchangeable, particularly in children with intracardiac shunts, where haemodynamic variability may affect the relationship between these parameters. This may have led to potential misclassification of disease severity. Children with Down syndrome in our cohort were younger, and age may act as a confounding factor; additionally, other potential influences on pulmonary hypertension severity, such as surgical status (repaired vs. unrepaired), oxygen saturation, and history of recurrent respiratory infections, were not accounted for in this analysis. Future studies should incorporate invasive haemodynamic assessment with right heart catheterisation to directly measure mPAP, or apply validated echocardiographic criteria specifically aligned with PASP-based severity definitions to ensure greater accuracy and comparability. Despite these limitations, the study provides valuable insights into the prevalence, severity, and risk factors for PH in children with CHD, particularly those with Down syndrome. A limitation of this study is that TAPSE was interpreted using adult-derived absolute cutoffs (<16 mm) rather than paediatric-specific Z-scores. Because right ventricular dimensions and function vary with age and body size, this may have led to misclassification of dysfunction in children, particularly younger patients or those with congenital heart disease. Future studies should use age- and body size-adjusted Z-scores for TAPSE to provide a more accurate assessment of right ventricular function in paediatric populations.

## Conclusions

Our findings indicate that children with Down syndrome and congenital heart disease, particularly those with complex lesions such as AVSD, exhibit a higher prevalence of severe pulmonary hypertension at a younger age among Bangladeshis. Given the cross-sectional nature of the study, causal relationships cannot be established; therefore, we recommended that future research focus on longitudinal studies to assess the progression of pulmonary hypertension and the impact of interventions in children with Down syndrome and congenital heart disease. Such studies would help clarify the disease patterns and inform strategies to improve long-term outcomes.
